# Supplementary material for: SUMO-mediated regulation of NLRP3 modulates inflammasome activity
Source: Nat Commun. 2018 Aug 1;9:3001. doi: 10.1038/s41467-018-05321-2 (PMC6070540; doi:10.1038/s41467-018-05321-2)
Supplement: Supplementary file 1 — Supplementary Information [file 41467_2018_5321_MOESM1_ESM.pdf]

Supplementary Information

SUMO-mediated Regulation of NLRP3 Modulates Inflammasome Activity

Barry et al., 2018

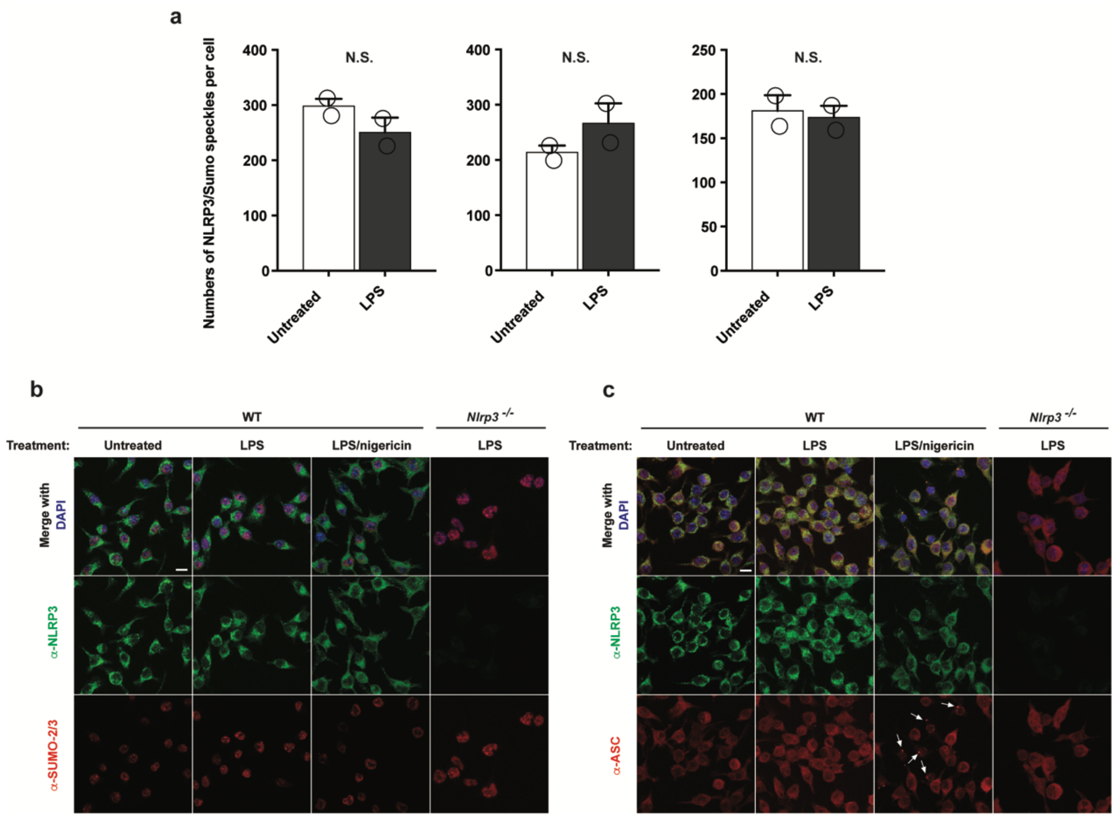

Supplementary Fig. 1: Immunofluorescence of endogenous NLRP3, SUMO-2/-3 and ASC. a Quantification of PLA SUMO:NLRP3 spots showing that treatment with LPS does not change sumoylation of NLRP3. The mean quantification of the PLA spots in 50 cells ±SD of technical repeats are shown from three independent experiments. Two-tailed Student's *t*-test was performed, N.S.= non-significant. **b, c** Confocal microscopy images showing NLRP3 (green) and SUMO-2/-3 (red) (b) and NLRP3 (green) and ASC (red) (c) staining. The same primary antibodies as used for PLA were employed. Nuclei were stained with DAPI (blue). Cells were treated as indicated. White arrows indicate ASC specks. Scale bar, 10 μm.

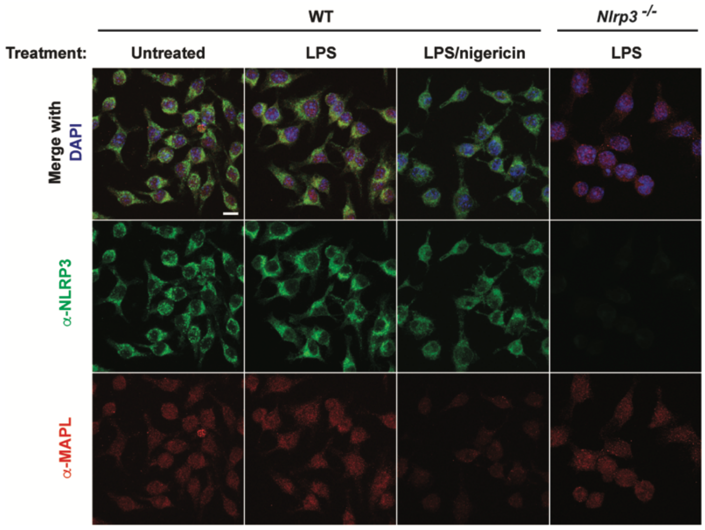

Supplementary Fig. 2: Immunofluorescence of endogenous NLRP3 and MAPL. Confocal microscopy images showing NLRP3 (green) and MAPL (red) staining. Nuclei were stained with DAPI (blue). The same primary antibodies as used for PLA were employed. Cells were treated as indicated. Scale bar, 10µm.

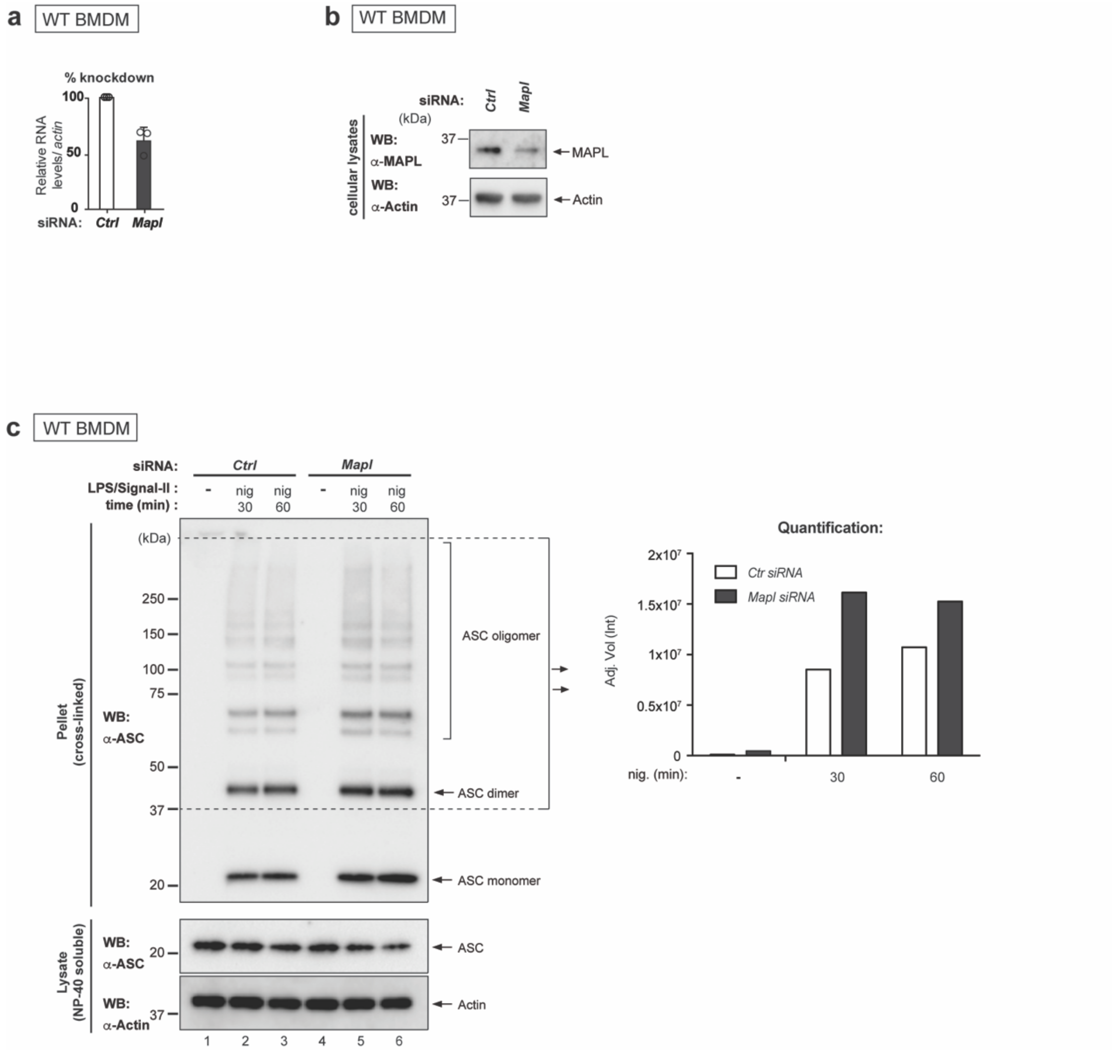

Supplementary Fig. 3: Depletion of MAPL affects endogenous association of NLRP3 with SUMO-2/3 and ASC, and leads to increased ASC oligomerisation. a, b Knockdown efficiency of *Mapl* by RNAi in BMDMs was determined by qRT-PCR. Individual data points from three biological repeats are plotted. Bars represent mean ±SEM (a) and Western blot (b). **c** *Mapl* knockdown leads to an increase in ASC oligomerisation upon nigericin treatment in *WT* BMDMs. 48 hrs post RNAi, cells were primed with LPS for 10 mins and stimulated for 0, 30, or 60 mins with nigericin. Cells were lysed in NP40 buffer and lysates were separated by centrifugation into NP-40-soluble (lysates) and NP-40-insoluble (pellets) fractions. Pellets were cross-linked for 30 mins prior immunoblot analysis. ASC oligomerisation smears were quantified using Image Lab Software (Bio-Rad) (graph).

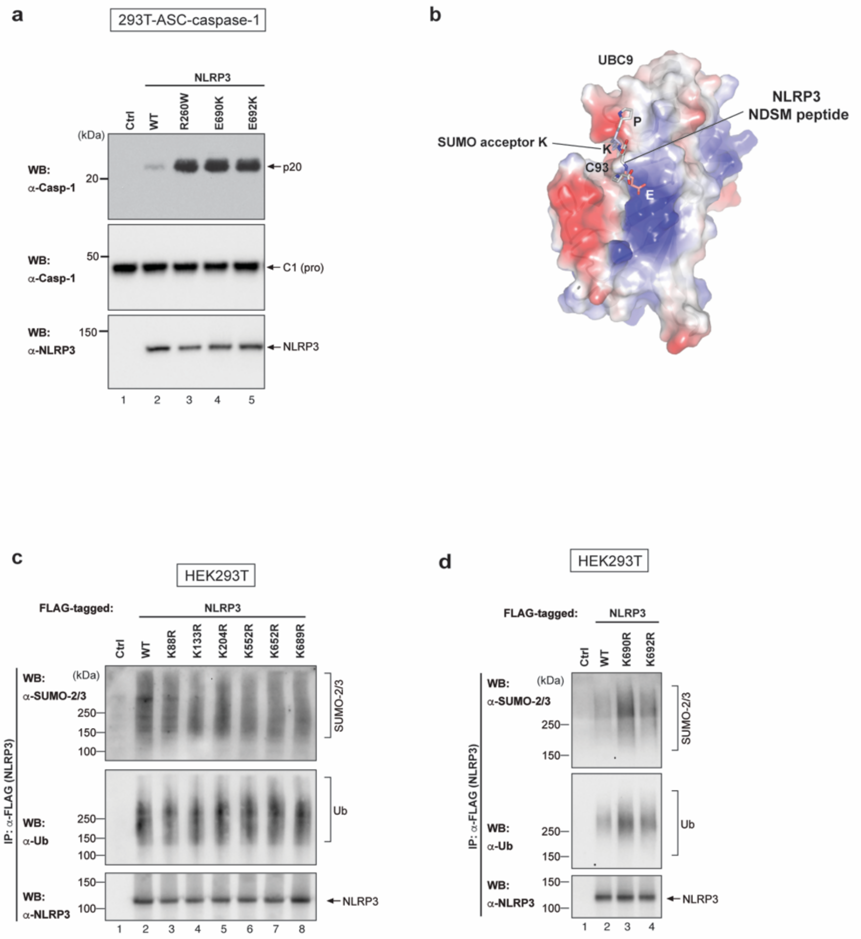

Supplementary Fig. 4: Mutation of K686 to R does not affect the global sumoylation of NLRP3. a Immunoblot analysis of inflammasome components (caspase-1, ASC and NLRP3) in cell lysates of HEK239T cells, stably expressing ASC and caspase-1, transfected with the indicated proteins. Caspase-1 maturation (p20 band) and NLRP3 modification was evaluated via western blot. **b** Model of surface representation of UBC9 bound to the SUMO consensus motif surrounding K689, colour coded by electrostatic potential of UBC9. *Red* to *blue* corresponds to negative to positive electrostatic potentials. **c, d** Sumoylation of NLRP3 in HEK293T cells transfected with the indicated constructs. FLAG-NLRP3 was purified under denaturing conditions and the presence of SUMO modification on NLRP3 was determined by immunoblot analysis of the elute.

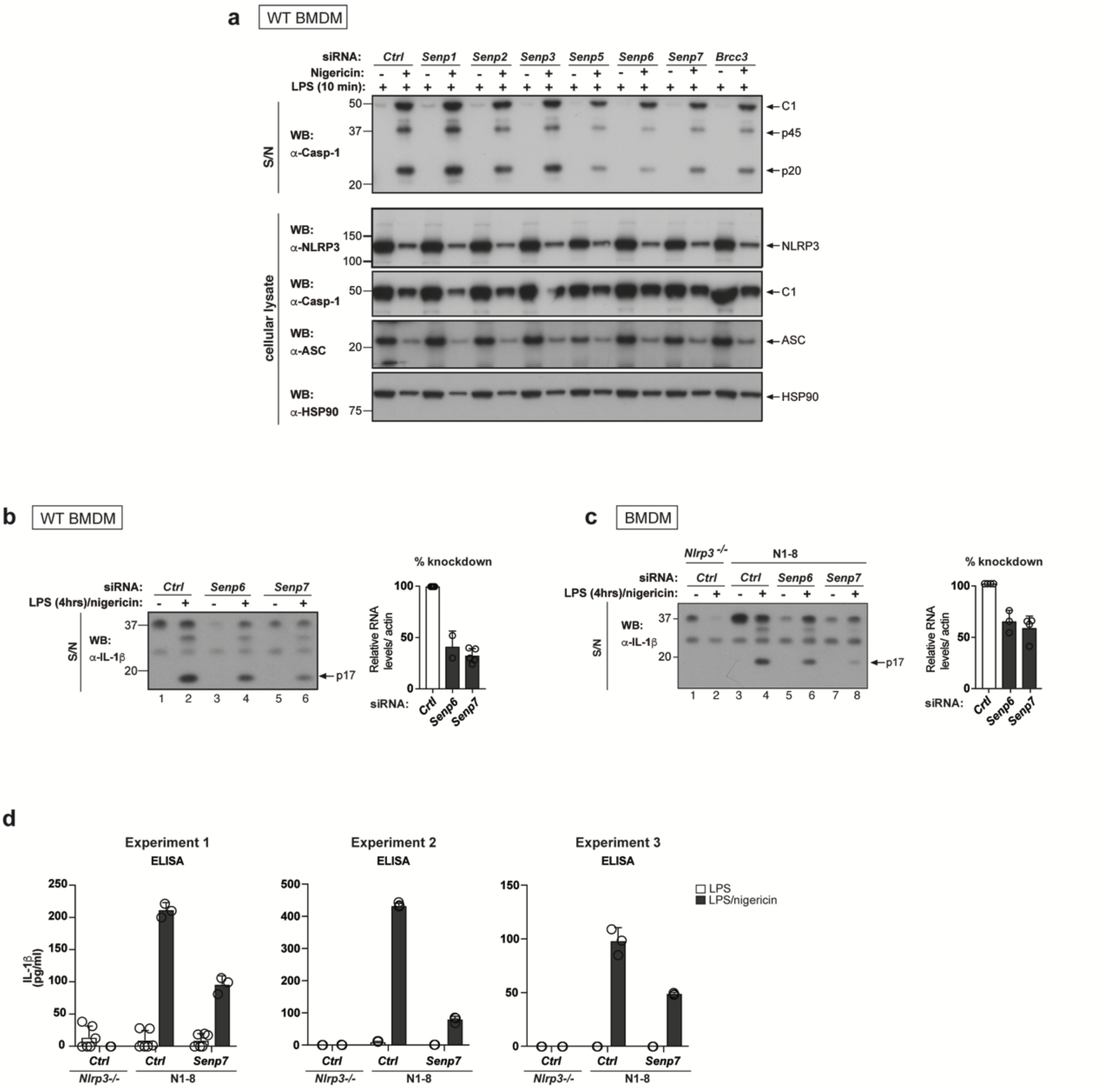

Supplementary Fig. 5 | Depletion of SENP6 and SENP7 reduces NLRP3 inflammasome activity. a The indicated BMDMs were transfected with the respective siRNAs and primed with LPS for 10 mins. Cells were subsequently stimulated with nigericin (90 mins). Representative immunoblot analysis of cleaved caspase-1 in cell supernatant (S/N) and ASC, caspase-1 and NLRP3 in the cellular lysate. **b**, **c** The indicated BMDMs were transfected with the respective siRNAs and primed with LPS for 4 hrs. Cells were subsequently stimulated with nigericin (90 mins). Representative immunoblot analysis of cleaved IL-1β in cell supernatant (S/N) is shown. The percentage of knockdown was quantified by qRT-PCR. Error bars represent the standard error of three individual experiments. (**d**) The concentration (pg/ml) of IL-1β from the three biological repeats (Fig. 6e) are plotted as individual graphs. The indicated BMDMs were treated with LPS for 4 hrs and stimulated with nigericin (90 mins). IL-1β levels in the cell supernatant were measured by ELISA. Individual data points from at least three technical repeats are plotted. Bars represent mean ±SD.

Supplementary Fig. 6 | Uncropped western blot images of the indicated Figures.

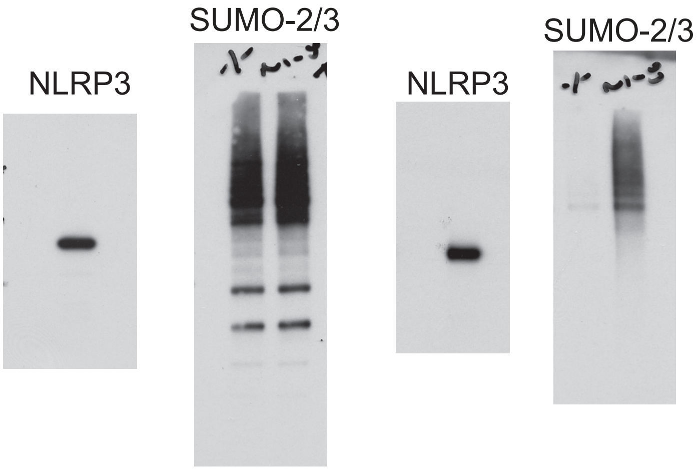

Figure 1c

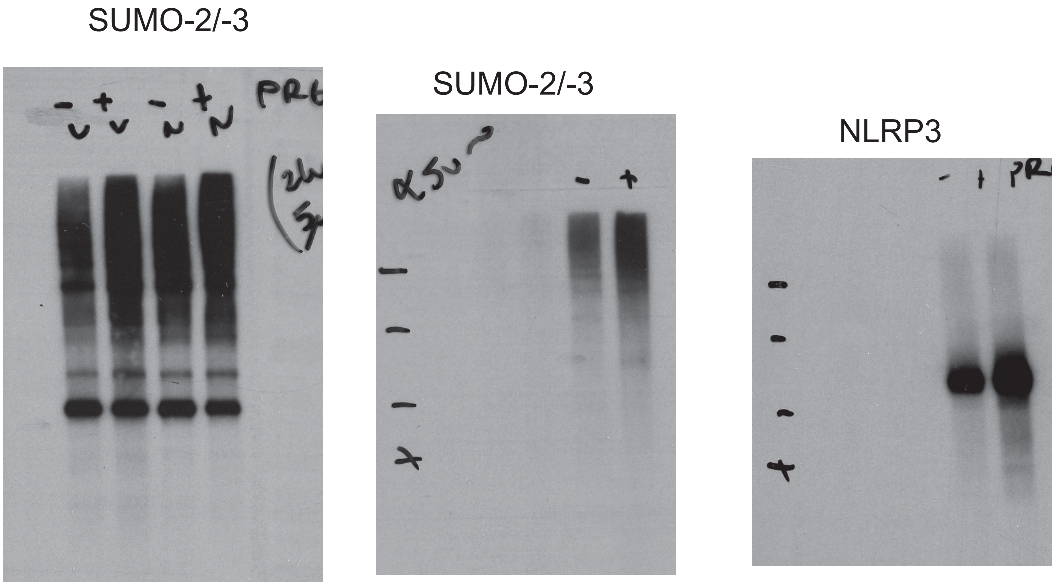

Figure 1d

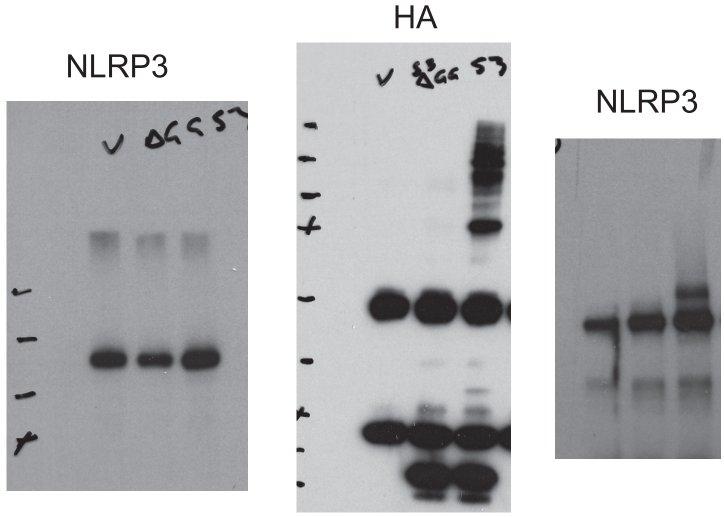

Figure 1e

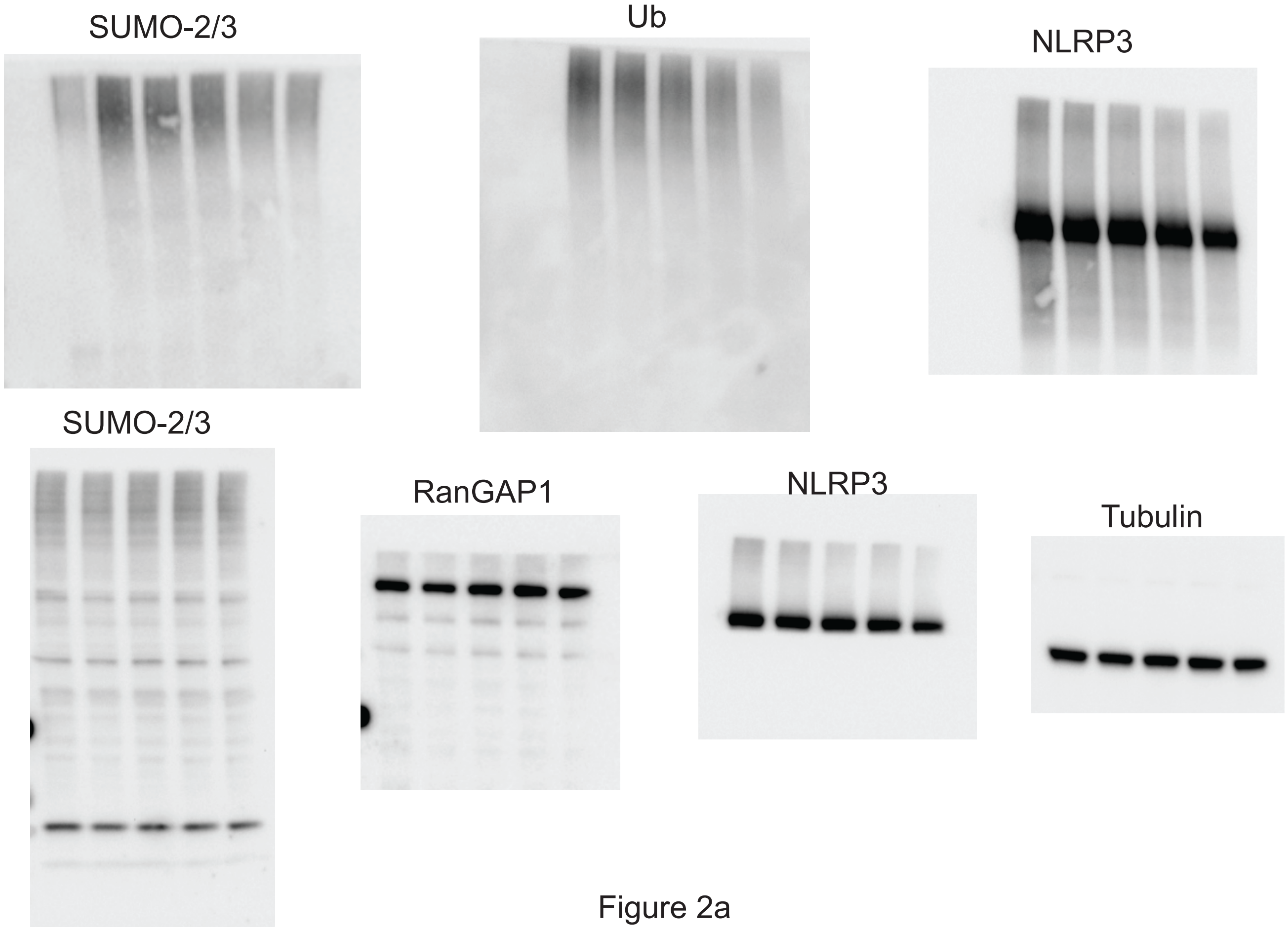

Figure 2a

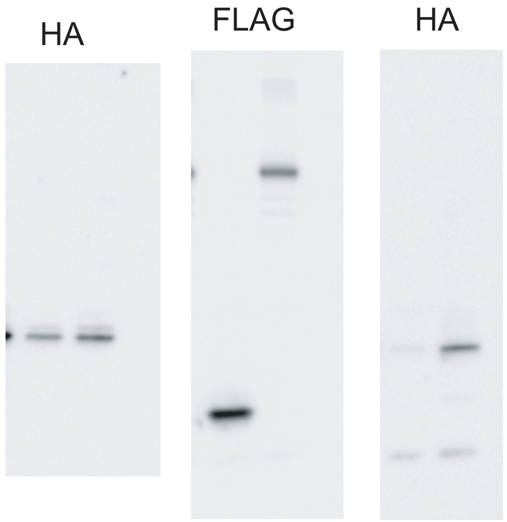

Figure 3a

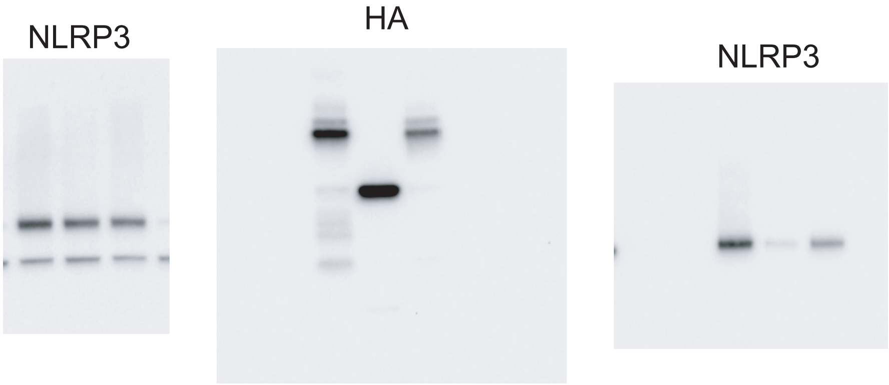

Figure 3b

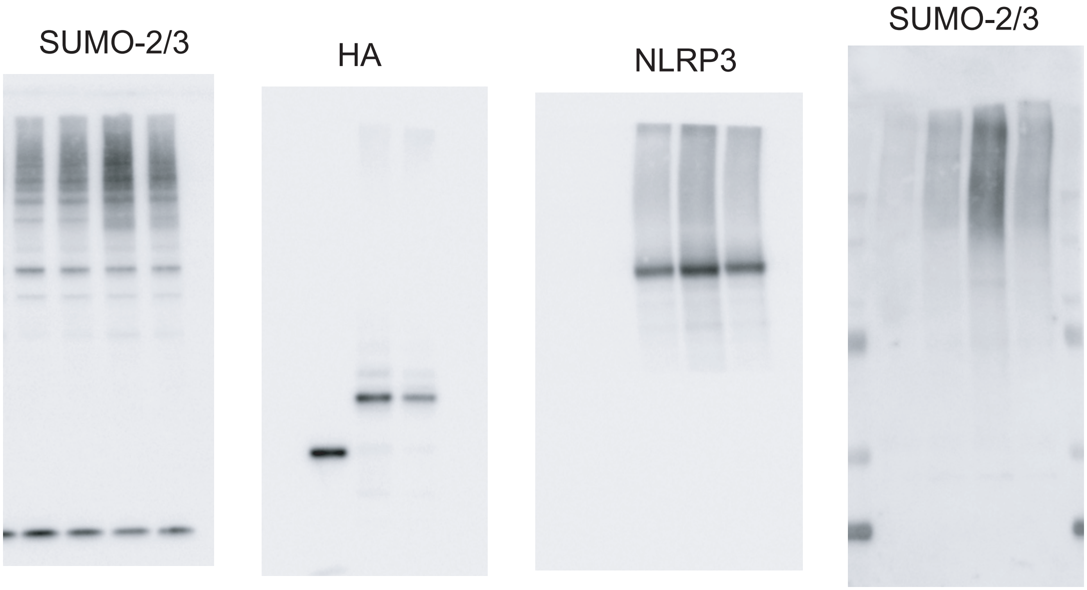

Figure 3c

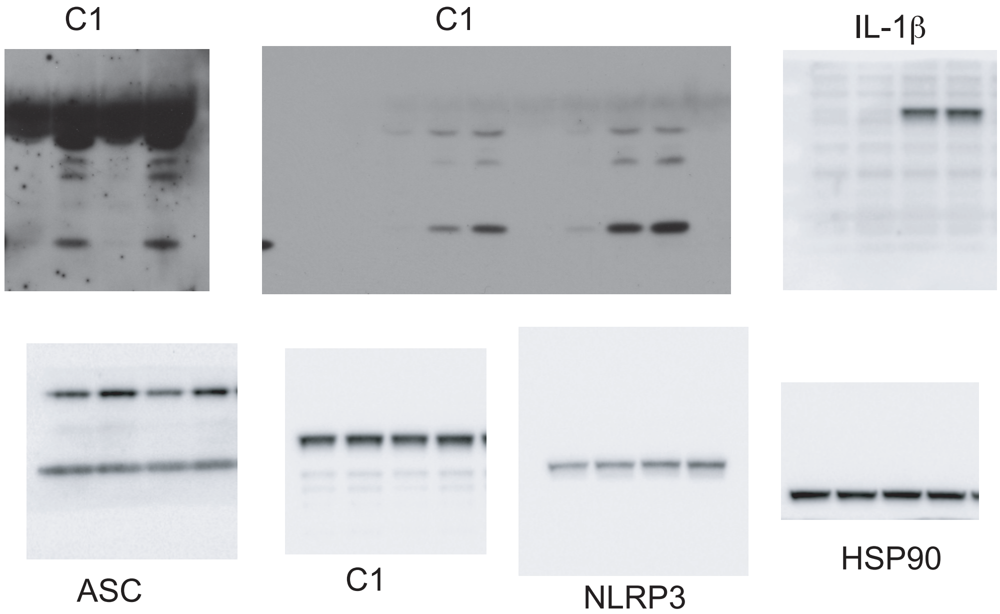

Figure 4c

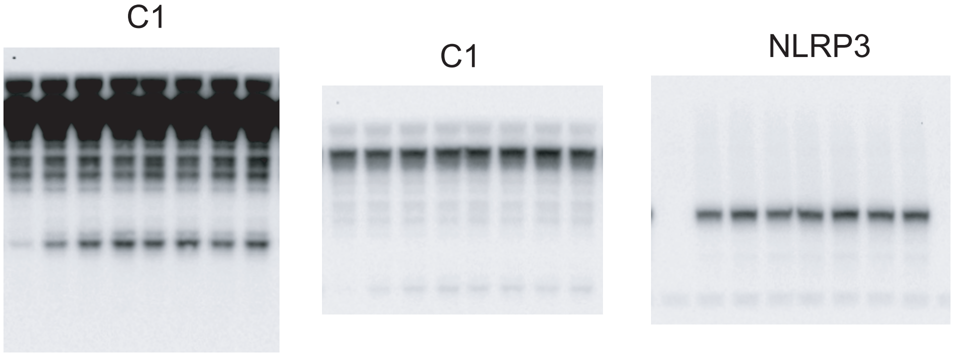

Figure 5a

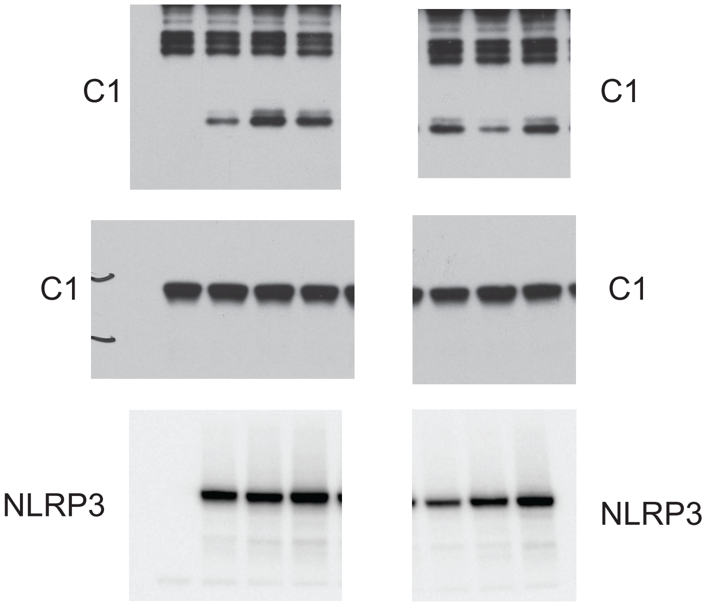

Figure 5b

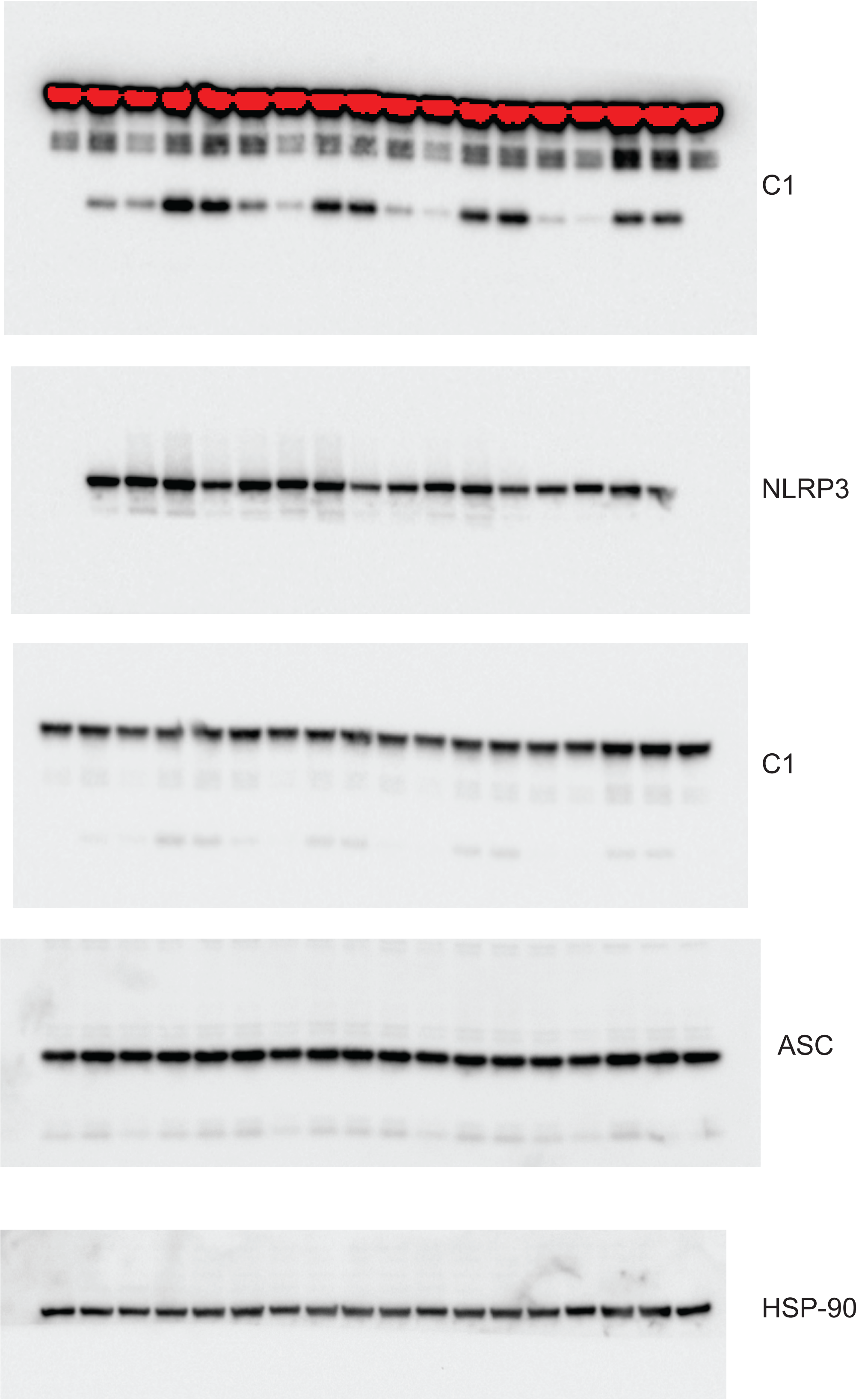

Figure 5c

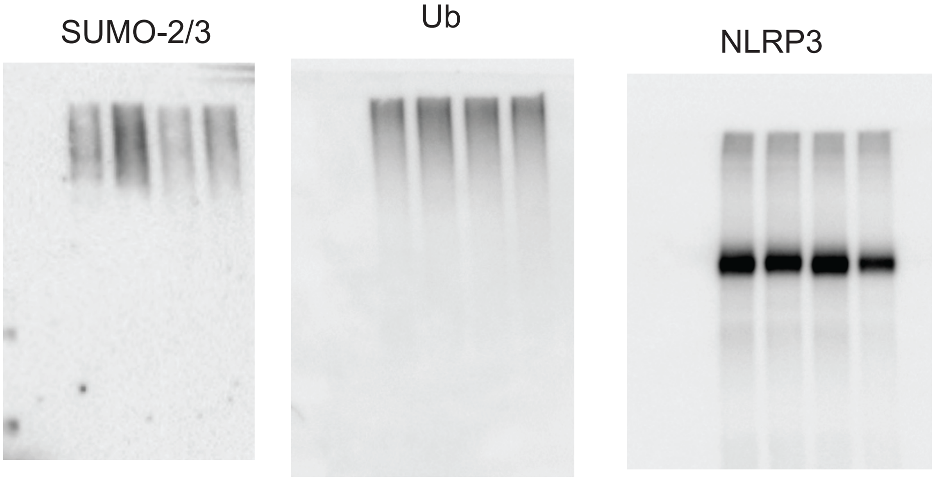

Figure 5f

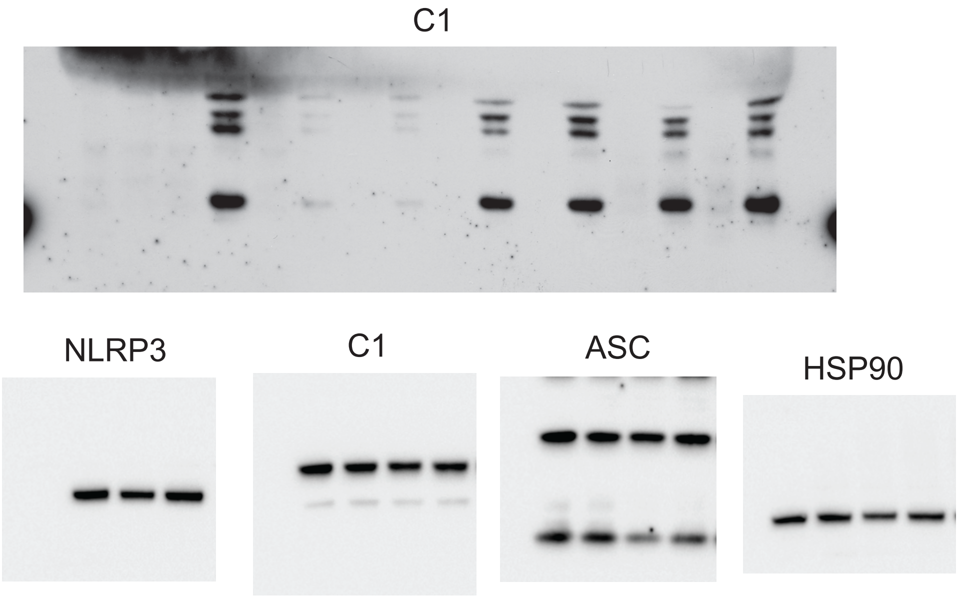

Figure 6a

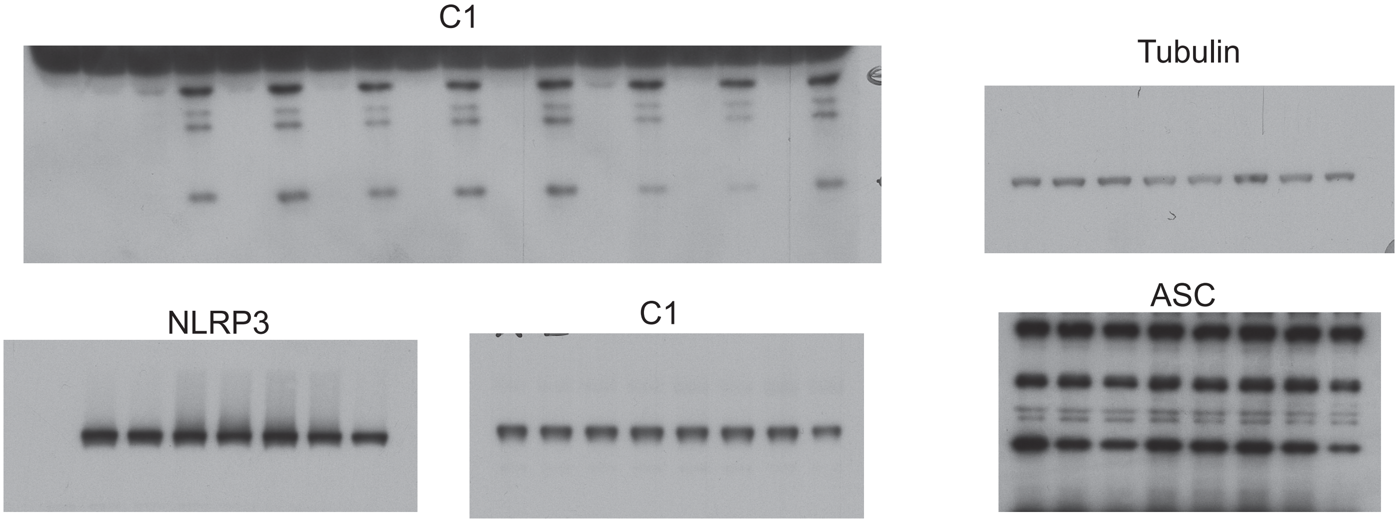

Figure 6b

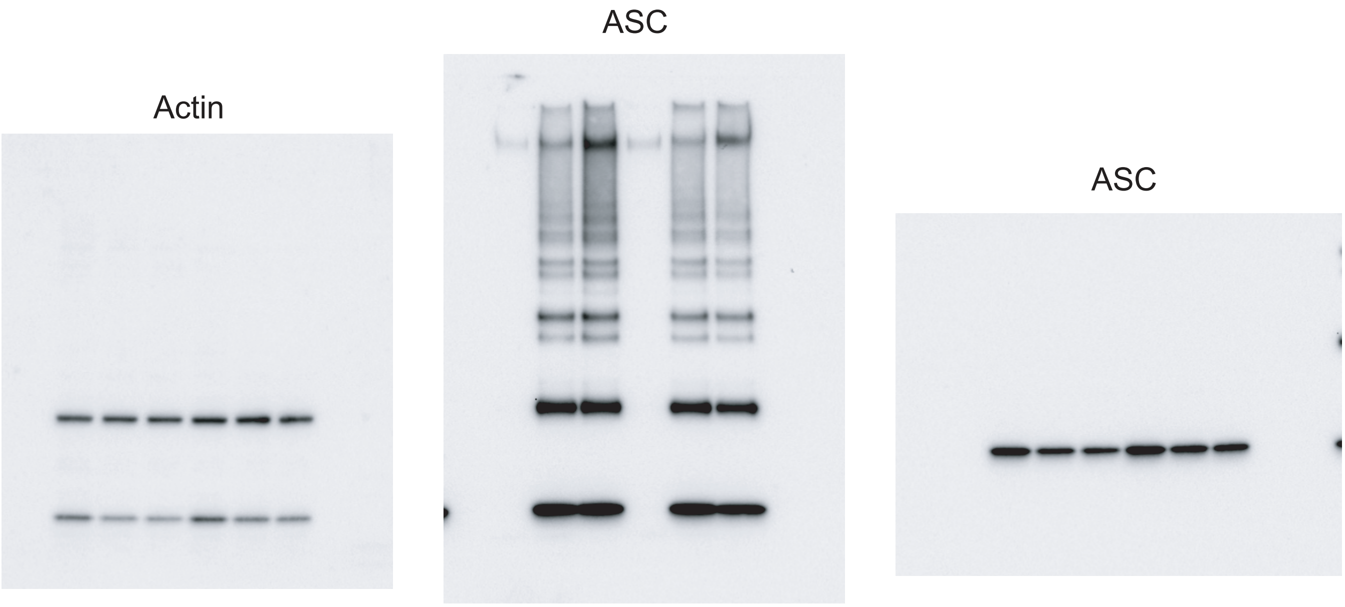

Figure 6c

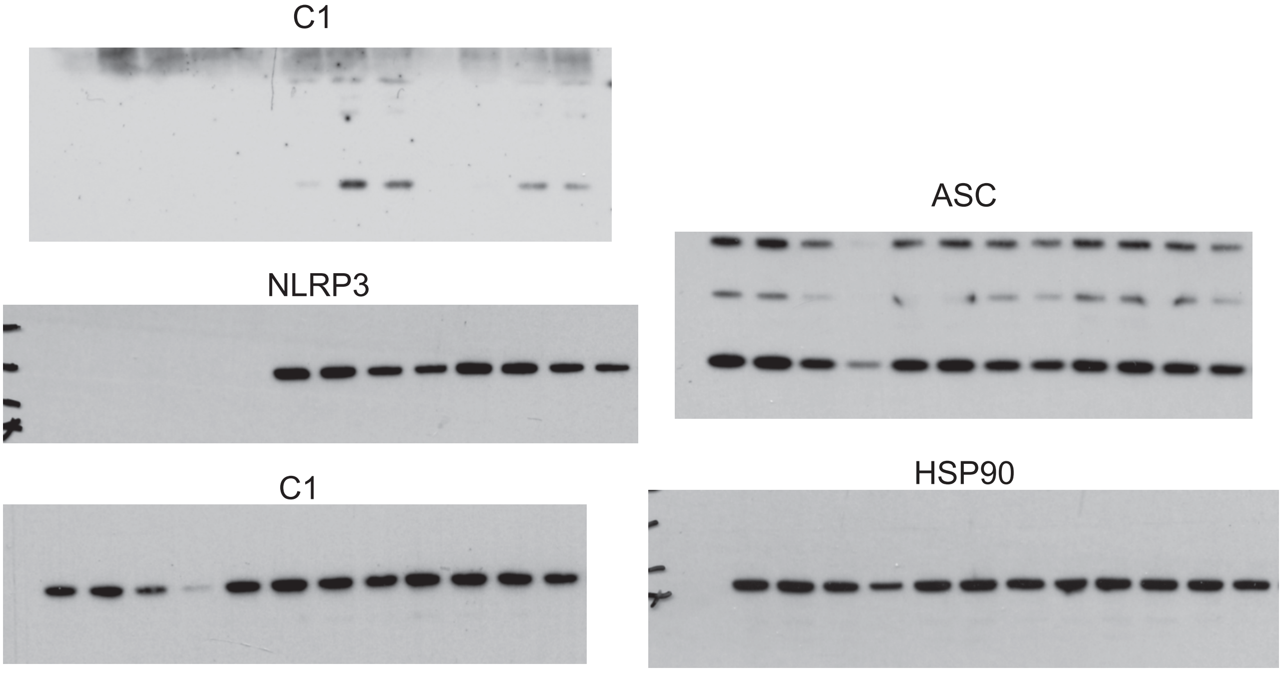

Figure 6d

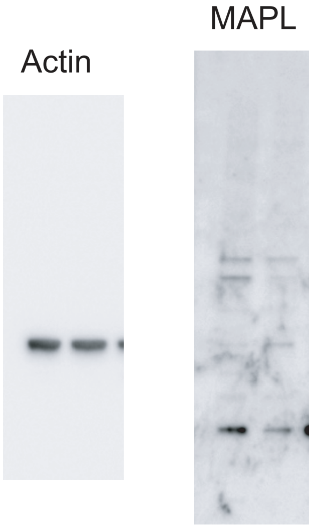

Supplementary Fig. 3b

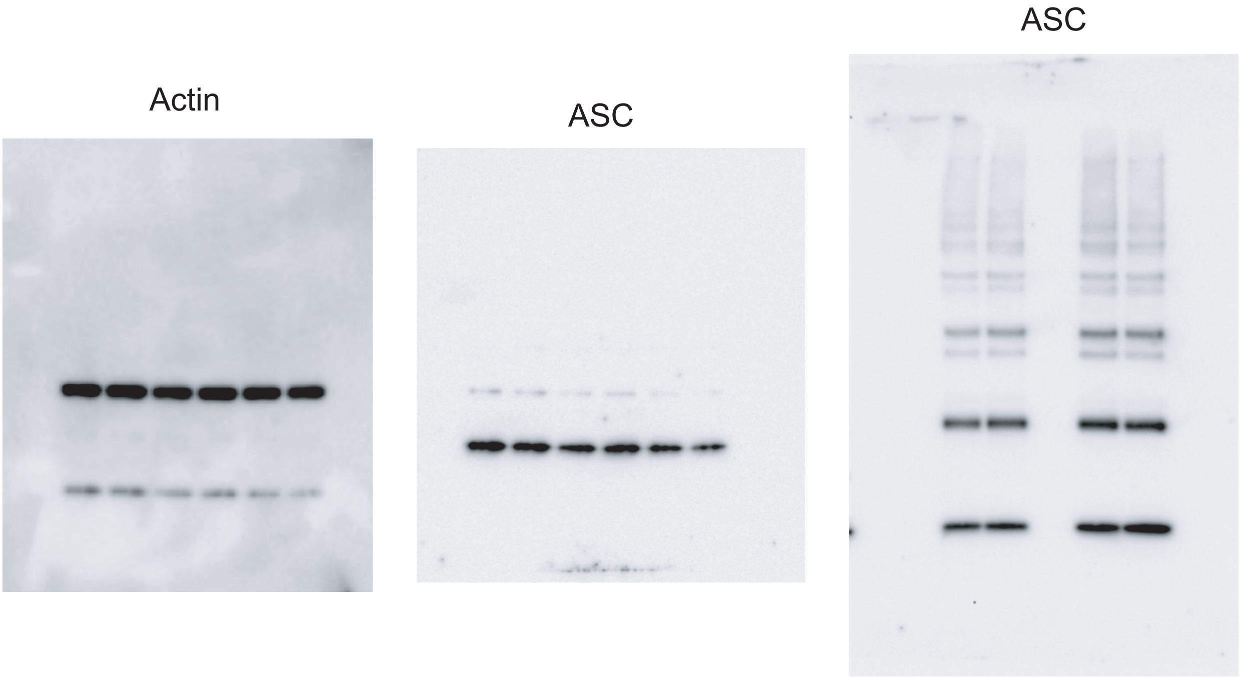

Supplementary Fig. 3c

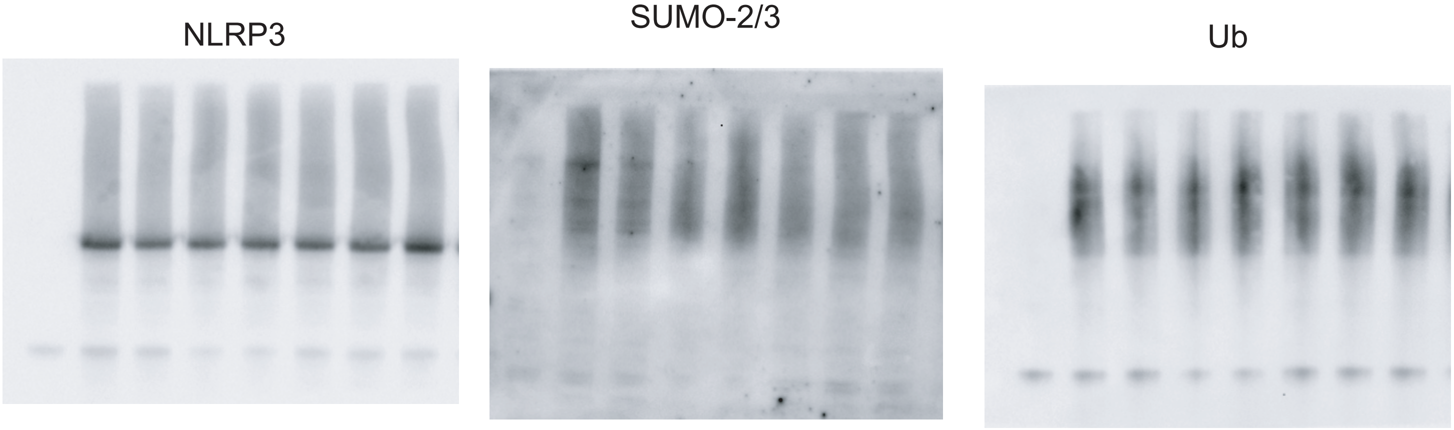

Supplementary Fig. 4a

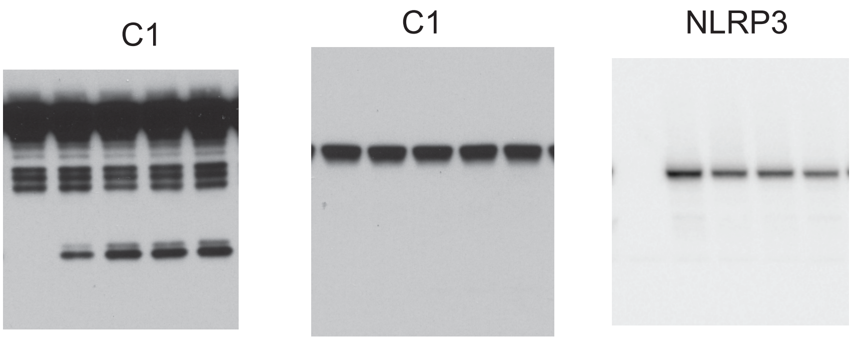

Supplementary Fig. 4c

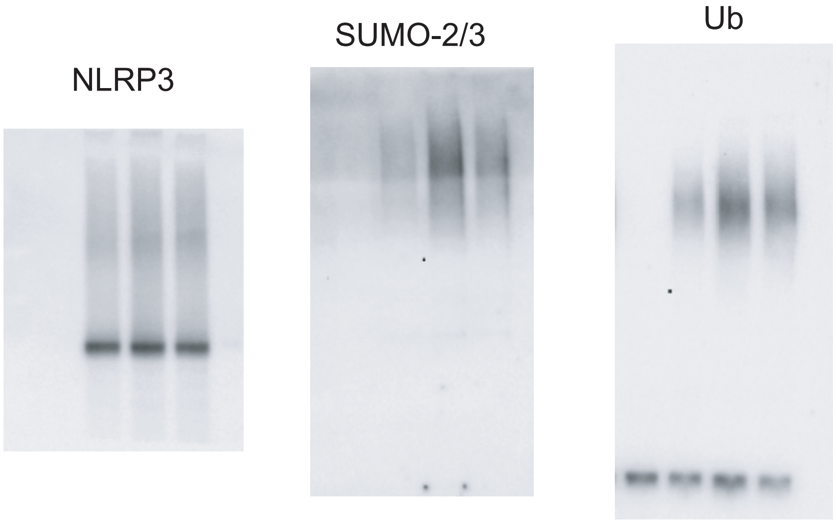

Supplementary Fig. 4d

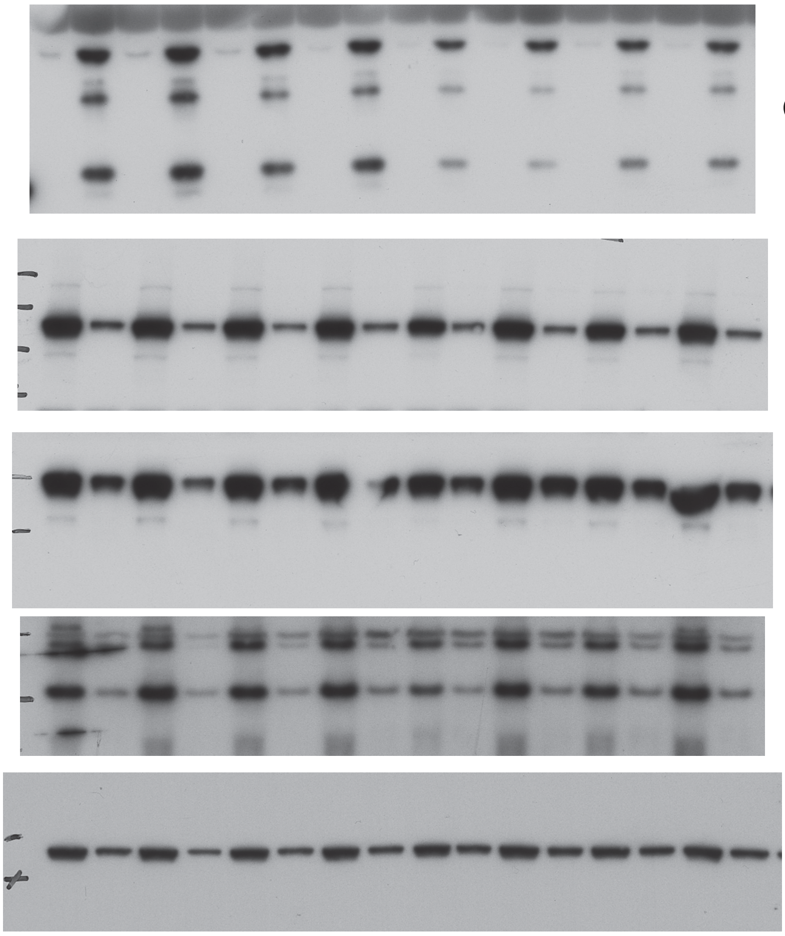

C1

NLRP3

C1

ASC

HSP90

Supplementary Fig. 5a

C1

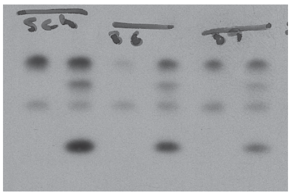

Supplementary Fig. 5b

C1

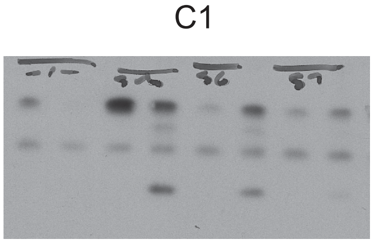

Supplementary Fig. 5c

Supplementary Table 1. Primers used for cloning

Name	Primer sequence (5'-3')
hNLRP3_K88R_for	GACCTTTATGAGAAAGCAAGAAGAGATGAGCCGAAGTGG
hNLRP3_K88R_rev	CCACTTCGGCTCATCTCTTCTTGCTTTCTCATAAAGGTC
hNLRP3_K133R_for	ATCTCTATTTGTAAAATGAGGAAAGATTACCGTAAGAAG
hNLRP3_K133R_rev	CTTCTTACGGTAATCTTTCCTCATTTTACAAATAGAGAT
hNLRP3_K204R_for	AGCCCCGTGAGTCCCATTAGGATGGAGTTGCTGTTT
hNLRP3_K204R_rev	AAACAGCAACTCCATCCTAATGGGACTCACGGGGCT
hNLRP3_K552R_for	GTTCCAGGGAGTCGTTTGAGGCTTCCCAGCCGAGACGTG
hNLRP3_K552R_rev	CACGTCTCGGCTGGGAAGCCTCAAACGACTCCCTGGAAC
hNLRP3_K652R_for	CATGGACTATTTCCCCAGGATTGAGATCAATCTCTC
hNLRP3_K652R_rev	GAGAGATTGATCTCAATCCTGGGGAAATAGTCCATG
hNLRP3_K689R_for	CATAACATGCCCAGGGAGGAAGAGGAGG
hNLRP3_K689R_rev	CCTCCTCTTCCTCCCTGGGCATGTTATG
hNLRP3_E690K_for	CTCCATAACATGCCCAAGAAGGAAGAGGAGGAGGAAAAG
hNLRP3_E690K_rev	CTTTTCCTCCTCCTCTTCCTTCTTGGGCATGTTATGGAG
hNLRP3_E692K_for	AACATGCCCAAGGAGGAAAAGGAGGAGGAAAAGGAAGGC
hNLRP3_E692K_rev	GCCTTCCTTTTCCTCCTCCTTTTCCTCCTTGGGCATGTT
pEF-HA-SUMO3-ΔGG_for	GGTACCATGTACCCATACGATGTTCCAG
pEF-HA-SUMO3-ΔGG_rev	CCGAATTCCTACGTCTGCTGCTGGAACACGTC
hMAPL_for	GGTACCATGGAGAGCGGAGGGCGGCCCTC
hMAPL_rev	GATATCCGCTGTTGTACAGGGGTATC
hMAPL_C339A_for	CCCAAGAAGTGCCCTATCGCCAGACAGGCGATCACCCGG
hMAPL_C339A_rev	CCGGGTGATCGCCTGTCTGGCGATAGGGCACTTCTTGGG
